# Growth and Reproduction of Glyphosate-Resistant and Susceptible Populations of *Kochia scoparia*

**DOI:** 10.1371/journal.pone.0142675

**Published:** 2015-11-18

**Authors:** Vipan Kumar, Prashant Jha

**Affiliations:** Montana State University-Bozeman, Southern Agricultural Research Center, Huntley, Montana, 59037, United States of America; Agriculture and Agri-Food Canada, CANADA

## Abstract

Evolution of glyphosate-resistant kochia is a threat to no-till wheat-fallow and glyphosate-resistant (GR) cropping systems of the US Great Plains. The *EPSPS* (5-enol-pyruvylshikimate-3-phosphate synthase) gene amplification confers glyphosate resistance in the tested *Kochia scoparia* (L.) Schrad populations from Montana. Experiments were conducted in spring to fall 2014 (run 1) and summer 2014 to spring 2015 (run 2) to investigate the growth and reproductive traits of the GR vs. glyphosate-susceptible (SUS) populations of *K*. *scoparia* and to determine the relationship of *EPSPS* gene amplification with the level of glyphosate resistance. GR *K*. *scoparia* inbred lines (CHES01 and JOP01) exhibited 2 to 14 relative copies of the *EPSPS* gene compared with the SUS inbred line with only one copy. In the absence of glyphosate, no differences in growth and reproductive parameters were evident between the tested GR and SUS inbred lines, across an intraspecific competition gradient (1 to 170 plants m^-2^). GR *K*. *scoparia* plants with 2 to 4 copies of the *EPSPS* gene survived the field-use rate (870 g ha^-1^) of glyphosate, but failed to survive the 4,350 g ha^-1^ rate of glyphosate (five-times the field-use rate). In contrast, GR plants with 5 to 14 *EPSPS* gene copies survived the 4,350 g ha^-1^ of glyphosate. The results from this research indicate that GR *K*. *scoparia* with 5 or more *EPSPS* gene copies will most likely persist in field populations, irrespective of glyphosate selection pressure.

## Introduction

*Kochia scoparia* (L.) Schrad. (kochia) is a monoecious, diploid (2n = 18) weed species, prevalent across the northern and central Great Plains of North America [1–3]. It is one of the most problematic, summer annual broadleaf weeds in cropland and non-cropland areas of this region. *K*. *scoparia* exhibits low seed dormancy, early seedling emergence, and rapid growth, and is tolerant to heat, salt, and drought stress [3–6]. A single plant can produce more than 100,000 seeds, and manifests a unique mechanism of seed dispersal through tumbling [3, 4]. During late fall, a mature plant breaks off at the base of the stem and tumbles across the landscape with the prevailing wind, dispersing seeds over a long distance [3, 4]. Because of the protogynous flowering, *K*. *scoparia* individuals can outcross, which contributes to the high genetic diversity within and among populations [7, 8]. *K*. *scoparia* populations resistant to photosystem II (PS II) inhibitors (atrazine), acetolactate synthase (ALS) inhibitors (sulfonylurea and imidazolinone), synthetic auxins (dicamba and fluroxypyr), and 5-enolpyruvylshikimate-3-phosphate synthase (*EPSPS*) inhibitor (glyphosate) have been reported in the US Great Plains [9].

*EPSPS* gene amplification is a novel mechanism of glyphosate resistance in weed species; first discovered in GR *Amaranthus palmeri* S. Wats. from Georgia [10]. In addition, *EPSPS* gene amplification has been reported in four other GR weed species, including *Amaranthus tuberculatus* (Moq.) J. D. Sauer [11], *Amaranthus spinosus* L. [12], *Lolium perenne* ssp. multiflorum [13], and recently in *K*. *scoparia* [14]. The massive amplification of the *EPSPS* gene in *A*. *palmeri* was likely mediated by transposable genetic elements, and amplified copies were dispersed throughout the genome [15]. However, it has recently been hypothesized that the *EPSPS* gene amplification in GR *K*. *scoparia* may possibly be mediated by unequal crossing over during meiosis, and the segregation of the amplified gene copies follows a single locus inheritance [16].

Novel herbicide-resistance conferring mutations may interfere with the normal plant function or metabolism, or potentially divert energy and resources away from growth and reproduction; hence, conferring fitness costs of the resistance alleles [17, 18]. Fitness cost is defined as the reduction of fitness caused by the negative effects of a resistance allele on vegetative growth, phenology, and fecundity characteristics of a herbicide-resistant (HR) relative to a herbicide-susceptible (HS) weed biotype [17, 18]. A fitness cost endowed by glyphosate resistance has been previously reported in GR *Lolium rigidum* Gaudin, wherein a reduced translocation of glyphosate was the resistance mechanism [19]. The GR individuals in that study produced fewer seeds than the susceptible plants, and there was a decline in the resistance phenotypic frequency from 45 to 11% after 3 years of no glyphosate use [19]. A GR *Ambrosia trifida* L. biotype from Indiana with a rapid necrosis response to glyphosate produced 25% less seed than a susceptible biotype, suggesting a fitness cost endowed by the glyphosate resistance trait [20]. On the contrary, a biotype of *A*. *trifida* from Wisconsin with no rapid necrosis response to glyphosate did not differ in growth and development compared to a susceptible biotype, but produced more seeds, 812 vs. 425 seeds plant^-1^ [21]. The *EPSPS* gene amplification mechanism of glyphosate resistance did not endow any fitness cost in GR *A*. *palmeri* biotypes from Georgia [22, 23]. Similarly, no difference in growth or fecundity was reported in GR *Conyza canadensis* L. populations from Indiana [24]. Nevertheless, a GR *C*. *canadensis* phenotype from California accumulated two times more biomass compared with a susceptible phenotype, when grown with or without competition with grapevine (*Vitis vinifera* L) [25]. This suggests that the fitness costs endowed by glyphosate resistance may vary with weed species, mechanism of glyphosate resistance, genetic background, and environment [22–24].

Glyphosate-resistant (GR) *K*. *scoparia* was first reported in 2007 from wheat fields in western Kansas [9]. Since then, it has been confirmed in nine US states and three Canadian provinces [9]. Recently, we reported *K*. *scoparia* populations with 4.6- to 11-fold levels of resistance to glyphosate from chemical fallow fields (wheat-chemical fallow rotation) in northern Montana [26]. *EPSPS* gene amplification (~ 4 to 10 relative copies of *EPSPS* gene) conferred resistance to glyphosate in those populations [27]. There is currently no published report on the relationship of *EPSPS* gene amplification with the growth and reproductive traits of GR *K*. *scoparia*. The objectives of this research were (1) to investigate the vegetative growth and reproductive traits of GR compared to glyphosate-susceptible (SUS) *K*. *scoparia* populations from Montana across an intraspecific competition gradient and (2) to determine the relationship of *EPSPS* gene amplification with the level of glyphosate resistance in GR *K*. *scoparia*.

## Materials and Methods

### Plant material

The glyphosate-resistant and susceptible *K*. *scoparia* seeds used in this study were collected from grower fields (private property). The growers/land owners provided the permission to enter their fields and collect the weed seeds. Since the authors work at the MSU Southern Agricultural Research Center at Huntley, MT, USA, no permission was needed to conduct the greenhouse and laboratory experiments. We also confirm that no field studies were conducted, and the research did not involve any endangered or protected species. Seeds of two GR *K*. *scoparia* populations (designated as JOP01 and CHES01) collected in the fall 2012 from two different chemical fallow fields (wheat-chemical fallow rotation) in the Liberty County, Montana, USA were used. The sampled fields had received three to four applications of glyphosate (each 870 g ae ha^-1^) per year for weed control during the chemical fallow period (preceding winter wheat) over more than 5 years. The populations (JOP01 and CHES01) were confirmed resistant to glyphosate [26]. The glyphosate-susceptible (SUS) *K*. *scoparia* population was collected from a wheat field with a similar herbicide use history in a 2 km vicinity of the fields where the two GR populations were collected.

Seeds of GR and SUS *K*. *scoparia* populations were sown on germination flats (53 × 35 × 10 cm) containing a commercial potting mix (VermiSoil, Vermicrop Organics, 4265 Duluth Avenue, Rocklin, CA) in a greenhouse at the Montana State University, Southern Agricultural Research Center (MSU-SARC) near Huntley, MT in the fall 2012. The greenhouse was maintained at 25/23 ± 3°C day/night temperatures, 80% relative humidity (RH), and 16 h photoperiod, supplemented with metal halide lamps (400 μmol m^-2^ s^-1^). *K*. *scoparia* plants (8- to 10-cm tall) from CHES01 and JOP01 populations (~150 plants per population) were treated with a discriminating dose of glyphosate at 1740 g ha^-1^ (twice the field-use rate of 870 g ha^-1^) (Roundup PowerMax, Monsanto Company, St Louis, MO). Glyphosate was applied using a stationary cabinet spray chamber equipped with an even flat-fan nozzle tip (TeeJet 8001XR, Spraying System Co., Wheaton, IL) calibrated to deliver 94 L ha^-1^ of spray solution at 276 KPa. The treated plants were returned to the greenhouse, and the survival was recorded 21 days after glyphosate treatment (DAT). *K*. *scoparia* plants from the CHES01 and JOP01 populations that survived 1740 g ha^-1^ of glyphosate, with < 50% injury and regrowth at 21 DAT were classified as GR. Out of 150 plants from the SUS population, none of them survived the 435 g ha^-1^ of glyphosate (half the field-use rate) at 21 DAT; confirming the susceptibility to glyphosate (data not shown).

### Development of GR and SUS inbred lines

GR (CHES01 and JOP01) and SUS *K*. *scoparia* individuals were developed by recurrent group selection under pollen isolation, rather than from a single seed, to prevent inbreeding depression, following a previously established method to develop dicamba-resistant *K*. *scoparia* inbreds [28]. Twenty phenotypically uniform plants of CHES01 or JOP01 population that survived the glyphosate rate of 1740 g ha^-1^ were used. A group of 2 to 3 plants were transplanted into a 20-L plastic pot containing the same potting mix as described above, and were covered with pollination bags (DelStar Technologies, Inc., 601 Industrial Drive, Middletown, DE). Fully matured seeds were collected and bulked separately for each population, and plants were subjected to two more generations of recurrent group selection (total of 3 generations) with the same dose of glyphosate. Seeds of the SUS *K*. *scoparia* were similarly obtained from a group of 20 phenotypically uniform SUS plants following three generations of restricted cross-pollination to prevent pollen contamination from other sources. None of the clones (shoot cuttings) derived from the SUS plants tested in each generation survived the 435 g ha^-1^ rate of glyphosate. Those relatively phenotypically uniform GR (CHES01 and JOP01) and SUS *K*. *scoparia* obtained after three generations of recurrent group selection were referred to as inbred lines, and were used for the subsequent experiments. The recurrent group selection technique to generate HR and HS inbred lines from field-collected weed populations has also been previously used to study fitness costs of HR alleles in multiple herbicide-resistant *Avena fatua* L., ALS inhibitor-resistant *K*. *scoparia*, and auxinic-herbicide resistant *K*. *scoparia* [29–31].

### Phenotypic characterization of glyphosate resistance

Greenhouse experiments were conducted at the MSU-SARC, Huntley, MT in the spring to fall 2014 (run 1) to investigate the growth and reproduction of selected GR *K*. *scoparia* inbred lines relative to the SUS inbred line across an intraspecific competition gradient. The experiments were repeated in summer 2014 to spring 2015 (run 2). The study was conducted in a completely randomized design with a factorial arrangement of treatments. Treatments included: three *K*. *scoparia* inbred lines (CHES01, JOP01, and SUS), three intraspecific plant densities of 1 (isolated plants), 4, and 8 *K*. *scoparia* plants pot^-1^, which corresponded to 1, 85 and 170 plants m^-2^ in the field. In each experimental run, there were 10 replicates with a total of 90 experimental units (20-L plastic pots). Out of the 10 replicated pots per treatment, 5 pots (designated set 1) were randomly selected for assessment of the vegetative growth parameters, and the remaining five pots (designated set 2) were used for assessing the reproductive parameters.

Seeds of each GR and SUS *K*. *scoparia* inbred line were separately sown on germination flats containing the same commercial potting mix described above. During pot filling, 5 g L^-1^ of slow release granular fertilizer (Osmocote Vegetable and Bedding [14–14–14], Scotts Company LLC, Marysville, OH) was added to the potting mix. *K*. *scoparia* seedlings (2- to 3-cm tall) were transplanted into the 20-L plastic pots, and were evenly spaced around the circumference (equidistant from the edge) of the pot. The greenhouse was maintained at similar temperature and photoperiod regimes as previously described. Plants were watered as needed to avoid moisture stress, and fertilized (Miracle-Gro water-soluble fertilizer [24–8–16], Scotts Miracle-Gro Products Inc., 14111Scottslawn Road, Marysville, OH) to maintain good growth.

#### *EPSPS* gene amplification

In both run 1 and run 2, from each actively growing young GR and SUS *K*. *scoparia* plants (8- to 10-cm tall) used for the evaluation of vegetative growth and reproductive traits (set 1 and 2), 100 mg of leaf tissue was sampled to determine the relative *EPSPS* genomic copy number using a quantitative real-time PCR (qPCR), following the previously established protocol [10, 14, 27]. The genomic DNA was extracted using a DNeasy plant mini kit (Qiagen) following the manufacturer’s protocol. DNA concentration was determined with a SmartSpec plus spectrophotometer (Bio-Rad Company), and the quality was ensured using 1% agarose gel electrophoresis. Only high quality (260/280 ratio of ≥ 1.8) DNA samples were utilized for determination of the *EPSPS* genomic copy number. The *ALS* gene was selected as a reference gene due to its stability across kochia populations [14, 27]. Primer sequences used for the *EPSPS* and *ALS* genes of *K*. *scoparia* have been previously reported. [14, 27]. The primer set for the *EPSPS* gene (EPSPF1: 5’-GGCCAAAAGGG CAATC GTGGAG-3’ and EPSPR1: 5’-CATTGCCGTTCCCG CGTTTCC-3’) produced approximately 102-bp product, and the *ALS* primer set (ALSF1: 5’-ATGCAGA CAATGT TGGATAC-3’ and ALSR1: 5’-TCAACCATCG ATA CGAACAT-3’) amplified 159-bp PCR products. For each primer pair, standard curves were developed using a 10-fold serial dilution of the high quality DNA. The primer efficiency was estimated to be 102% for *EPSPS* and 101% for *ALS*. The qPCR reaction contained 2.5 μl of DNA (2 ng ul^-1^) template, 1X Perfecta SYBR green supermix, 250 nM of each of forward and reverse primers. The reaction efficiency for qPCR was estimated to be 101% (R^2^ of 0.99 and a slope of -3.291). Each qPCR reaction was performed in triplicates, with a final reaction volume of 20 μl on a Bio-Rad 96-well PCR plate. The qPCR assay was performed on CFX Connect Real-Time PCR detection system (Bio-Rad Company) under following conditions: 98°C for 2 min, 40 cycles of 98°C for 5 s, and 60°C for 30 s, followed by a melt-curve analysis. A negative control consisting of 250 nM of each primer, SYBR green supermix, and deionized water with no DNA template was included. The *EPSPS* genomic copy number relative to *ALS* gene was quantified by ΔC_T_ method (ΔC_T_ = C_T_, *ALS*–C_T_, *EPSPS*) [10, 14]. The relative increase in the *EPSPS* gene copy number was calculated as 2^ΔCT^.

#### Vegetative growth

Growth measurements including plant height (measured from the terminal leaf to the base of the plant), plant width (maximum canopy diameter), and number of primary branches plant^-1^ were recorded at the maximum vegetative stage (at the first visible sign of flower initiation). Plants (set 1) were then harvested at the soil level, and leaves were manually separated from the branches and stem tissues. The total leaf area plant^-1^ was determined using a leaf area meter (LI-COR 3000, LII-COR, Inc., Lincoln, NE). The aboveground vegetative biomass (leaves, stems/branches) was oven dried at 60°C for 5 days, and the aboveground biomass plant^-1^ was determined.

#### Fecundity

For fecundity assessments, the five replicated plants per treatment (set 2) were individually covered with pollination bags to prevent cross pollination, before flower initiation. Plants were manually harvested at maturity (when plants were fully brown in appearance), and placed in separate paper bags. Seeds were manually separated from the inflorescence, and air dried at room temperature for 2 weeks; the coarse debris was removed using a 2-mm mesh size sieve, and the small debris was removed with an air-propelled column blower (Seedburo Equipment Co., 2293 S. Mt. Prospect Road, Des Plaines, IL). The aboveground biomass was determined as described above. The mass of 1000 seeds and total seed mass plant^-1^ were determined to estimate the seed production per plant. The harvest index was calculated as a ratio of total seed mass to total aboveground biomass per plant [32].

#### Seed viability and radicle length

Laboratory experiments were conducted with fully matured seeds harvested in fall 2014 (run 1) and spring 2015 (run 2) in a completely randomized design with four replications (50 seeds per petri dish per replication). A subsample of 200 seeds produced by each inbred line per intraspecific density treatment (set 2) was randomly selected. Seeds were placed between two layers of filter papers (Whatman^®^, Grade 2, Sigma-Aldrich Inc., St. Louis, MO 63178, USA) in 10-cm diameter petri dishes (Sigma-Aldrich) moistened with 10 ml of deionized water. Because light is not needed for seed germination of *K*. *scoparia* [33], all petri dishes were wrapped in aluminum foil, and placed in the dark in an incubator (VMR International, Sheldon Manufacturing Inc., Cornelius, OR). The incubator was set to a constant temperature of 24°C, which is considered optimum for germination of *K*. *scoparia* seeds [34]. Radicle lengths of germinating seedlings were recorded after 24 h of incubation. The number of germinated seeds was counted daily until 15 days after incubation. The non-germinated seeds were tested for viability using a 1% w/v tetrazolium chloride solution [35]. Seeds were considered germinated when the radicles emerged and the tip of the radicle was uncoiled [36]. Seed viability was estimated as the percentage of total seeds that germinated plus those tested positive in the tetrazolium chloride assay.

### Relationship of *EPSPS* genomic copy number and resistance

Greenhouse experiments were conducted in spring 2014 (run 1) and repeated in summer 2014 (run 2). After determining the *EPSPS* genomic copy number described above, two set of clones were prepared from the GR (CHES01 and JOP01) and SUS *K*. *scoparia* plants grown for the assessment of reproductive parameters (set 2). Two uniform 5-cm long sections from a lateral branch of each plant were precisely cut, when the plants were 80 cm tall. After removing leaves from the base, those sections were re-cut at an angle to enhance the uptake of the rooting hormone [RootBoost (Indole-3-butyric acid 0.1%) -Rooting Hormone, TechPac LLC. 2030 Powers Ferry Road, Ste. 370 Atlanta, GA). Cuttings were dipped into the rooting hormone, and planted in plastic pots (6 × 6 × 10 cm) containing the same potting mix as previously described. A total of 150 clones per *K*. *scoparia* inbred line (SUS, CHES01, or JOP01) were prepared, and subdivided into two sets. One set of actively growing 8- to 10-cm-tall *K*. *scoparia* clones were treated with 870 g ha^-1^ (field-use rate) of glyphosate. The second set of clones were treated with glyphosate at 4,350 g ha^-1^ (five times the field-use rate). Ammonium sulfate (AMS) at 2% (w/v) was included with all glyphosate treatments. After the glyphosate treatment, clones were returned to the greenhouse, watered to avoid moisture stress, and fertilized. Percent injury from glyphosate at 870 and/or 4350 g ha^-1^ was visually assessed on a scale of 0 (no injury) to 100 (complete plant death) at 21 DAT.

### Statistical analyses

All data were subjected to ANOVA using PROC MIXED in SAS (SAS Institute Inc., Cary, NC) to test the significance of experimental run, inbred line, and treatment (plant density in the vegetative and reproductive growth study or glyphosate dose in the glyphosate resistance experiment) and their interactions. Data were checked to test normality of residuals and homogeneity of variance using PROC UNIVARIATE and PROC GLM in SAS (Statistical Analysis Systems^®^, version 9.2, SAS Institute Inc., SAS Campus Drive, Cary, NC). Means were separated using Fisher’s protected LSD test at P < 0.05. Pearson’s correlation analyses were performed to test the relationship between the *EPSPS* gene amplification and vegetative growth parameters (plant height, width, number of primary branches, leaf area, and aboveground biomass) or reproductive parameters (harvest index and 1000-seed mass) of GR and SUS *K*. *scoparia* at each intraspecific density treatment. A correlation analysis was also performed to determine the degree of relationship between the *EPSPS* gene copy number and percent injury with glyphosate at 870 or 4,350 g ha^-1^ rate. The Pearson’s correlation coefficient (*r*) was estimated using the CORR Procedure in SAS. A simple linear regression analysis was also performed using PROC REG in SAS to quantify the relationship between *EPSPS* gene copy number and percent injury.

## Results

### Phenotypic characterization of glyphosate resistance

#### Correlation of *EPSPS* gene amplification with growth and reproductive traits

**Fig 1:**
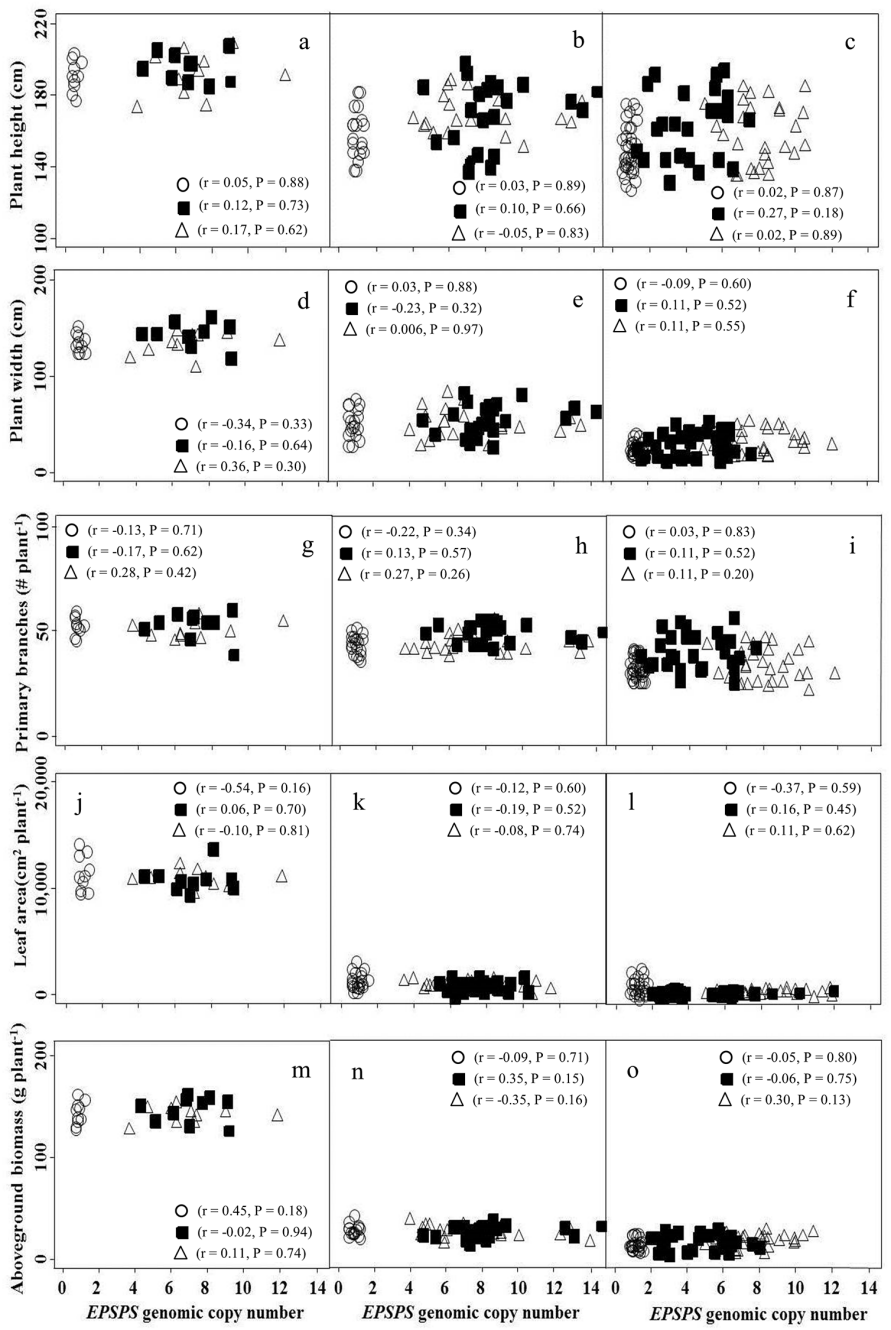
Correlation of vegetative growth traits with *EPSPS* genomic copy number in SUS (open circles), CHES01 (filled squares), and JOP01 (open triangles) *K*. *scoparia* inbred lines grown under densities of 1 (a, d, g, j, m), 85 (b, e, h, k, n), and 170 (c, f, i, l, o) plants m^-2^. Sample size was pooled from two experimental runs (n = 10, 40, and 80 for densities of 1, 85, and 170 plants m^-2^, respectively). For each pairwise combination of traits and *EPSPS* genomic copy number, P values from the correlation analysis and Pearson’s correlation coefficients (*r*) are shown. CHES01 and JOP01 are the glyphosate-resistant *K*. *scoparia* inbred lines and SUS is the glyphosate-susceptible inbred line.

**Fig 2:**
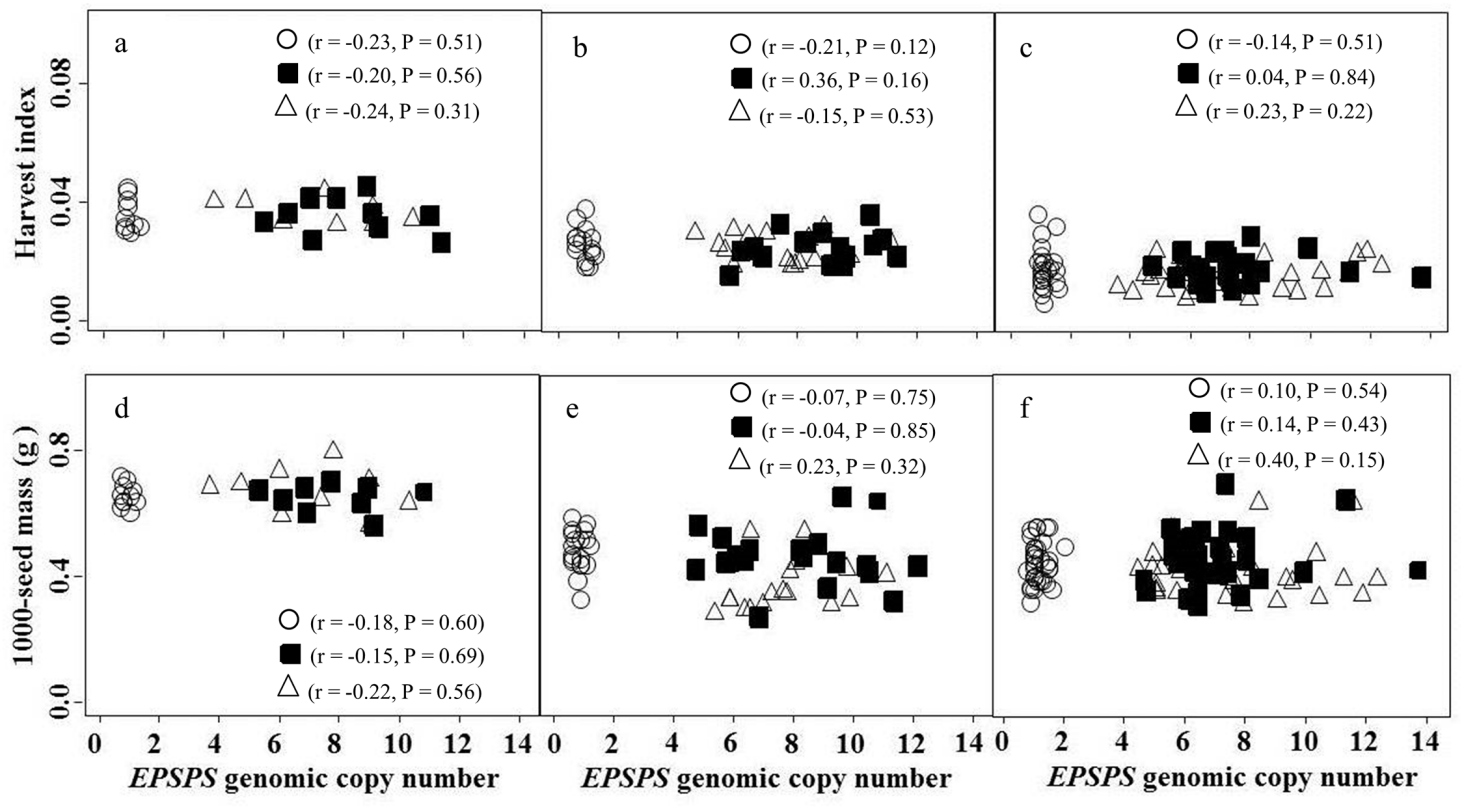
Correlation of reproductive traits with *EPSPS* genomic copy number in SUS (open circles), CHES01 (filled squares), and JOP01 (open triangles) *K*. *scoparia* inbred lines grown under densities of 1 (a, d), 85 (b, e), and 170 (c, f) plants m^-2^. Sample size was pooled from two experimental runs (n = 10, 40, and 80 for densities of 1, 85, and 170 plants m^-2^, respectively). For each pairwise combination of traits and *EPSPS* genomic copy number, P values from the correlation analysis and Pearson’s correlation coefficients (*r*) are shown. CHES01 and JOP01 are the glyphosate-resistant *K*. *scoparia* inbred lines and SUS is the glyphosate-susceptible inbred line.

Based on the nonsignificant experimental run by treatment interaction (P = 0.631), data were pooled from the two runs. The *EPSPS* gene copy number ranged from 2 to 14 among individuals of CHES01 and from 3 to 13 among individuals of JOP01 GR inbred lines. The field-collected individuals from those GR *K*. *scoparia* biotypes had 6- to 10-fold amplification of the *EPSPS* gene [27]. In contrast, the SUS individuals had a single copy of the *EPSPS* gene. The quantitative changes in growth and reproductive traits of GR vs. SUS plants were assessed across the *EPSPS* gene copy numbers. The *EPSPS* gene copy number did not correlate with vegetative growth parameters, including plant height, canopy width, primary branches, total leaf area, and shoot biomass of GR or SUS *K*. *scoparia*, whether the plants were grown at intraspecific densities of 1 (Fig 1a, 1d, 1g, 1j, and 1m), 85 (Fig 1b, 1e, 1h, 1k, and 1n), or 170 (Fig 1c, 1f, 1i, 1l, and 1o) plants m^-2^. Similarly, there was no significant correlation between the *EPSPS* gene copy number and harvest index or 1000-seed mass, regardless of the competition gradient (Fig 2a, 2d, 2b, 2e, 2c, and 2f). Thus, the 10- to 14-fold or 2- to 5-fold *EPSPS* gene amplification had no association with any of the tested growth or reproductive parameters of GR *K*. *scoparia* inbred lines compared with the SUS inbred line with a single copy of the *EPSPS* gene along the competition gradient.

#### Vegetative growth

Data were pooled across runs on the basis of a nonsignificant experimental run by treatment interaction (P = 0.521). Results from the ANOVA indicated that the vegetative growth parameters tested did not differ among the GR or SUS *K*. *scoparia* inbred lines (P = 0.142). Only the main effect of *K*. *scoparia* density was significant (P < 0.001). With an increase in intraspecific competition (density), there was a decrease in vegetative growth of plants. Increasing the density from 1 to 170 plants m^-2^ reduced plant height by 19%, plant width by 72% and number of primary branches by 36% when averaged across the inbred lines (Tables 1 and 2). Total leaf area of *K*. *scoparia* plants grown under intraspecific densities of 85 and 170 plants m^-2^ were 88 and 95% less compared with the plants grown without competition (density of 1 plant m^-2^), respectively. Similarly, increasing the intraspecific competition from 1 to 170 plants m^-2^ reduced shoot biomass per plant by 83%, irrespective of GR or SUS inbred line.

**Table 1:** Effect of intraspecific competition on plant height, plant width, and primary branches of glyphosate-susceptible (SUS) and glyphosate-resistant (CHES01 and JOP01) *K*. *scoparia* inbred lines averaged over two runs.^a^ ^,^ ^b^

	Plant height (cm)				Plant width (cm)				Primary branches (# plant^-1^)			
Density (plants m^-2^)	SUS	CHES01	JOP01	Pooled	SUS	CHES01	JOP01	Pooled	SUS	CHES01	JOP01	Pooled
**1**	197 (3.6)	196 (3.5)	196 (3.1)	196 a	142 (2.3)	145 (2.4)	143 (2.1)	143 a	54 (2.0)	56 (2.0)	56 (2.1)	55 a
**85**	165 (1.6)	170 (1.6)	166 (1.7)	167 b	55 (0.8)	59 (0.9)	55 (3.7)	56 b	44 (0.9)	45 (0.9)	43 (1.0)	44 b
**170**	158 (0.8)	162 (0.6)	160 (1.0)	160 c	39 (0.7)	40 (2.8)	39 (3.7)	39 c	33 (0.6)	37 (0.7)	35 (0.7)	35 c

^a^ Values in parenthesis represent standard error of the mean.

^b^ Means within a pooled column with similar letters are not different based on Fisher’s protected LSD test at P < 0.05.

**Table 2:** Effect of intraspecific competition on total leaf area and aboveground biomass of glyphosate-susceptible (SUS) and glyphosate-resistant (CHES01 and JOP01) *K*. *scoparia* inbred lines averaged over two runs^a^ ^,^ ^b^.

Density (plants m^-2^)	Leaf area (cm^2^ plant^-1^)				Aboveground biomass (g plant^-1^)			
	SUS	CHES01	JOP01	Pooled	SUS	CHES01	JOP01	Pooled
**1**	11,820 (272)	11,806 (266)	11,623 (271)	11,750 a	150 (1.7)	149 (1.5)	151 (1.8)	150 a
**85**	1,454 (202)	1,413 (209)	1,445 (203)	1,437 b	32 (0.8)	34 (0.6)	29 (0.9)	32 b
**170**	576 (58)	548 (87)	555 (91)	560 c	22 (0.7)	27 (0.8)	25 (0.7)	25 c

^a^ Values in parenthesis represent standard error of the mean.

^b^ Means within a pooled column with similar letters are not different based on Fisher’s protected LSD test at P < 0.05.

#### Fecundity

The effect of experimental run by treatment was not significant (P = 0.325). Similarly to vegetative growth parameters, fecundity and seed mass were influenced only by the *K*. *scoparia* density (P < 0.001). Averaged across the inbred lines, plants grown under intraspecific densities of 85 and 170 plants m^-2^ had 32 and 55% less harvest index, respectively, compared with the plants grown without competition (Table 3). Similarly, an increase in *K*. *scoparia* density from 1 to 85 plants m^-2^ caused reduction in 1000-seed mass; however, no further decline in seed mass was observed by increasing the density to 170 plants m^-2^ (Table 3).

**Table 3:** Effect of intraspecific competition on reproductive traits of glyphosate-susceptible (SUS) and glyphosate-resistant (CHES01 and JOP01) *K*. *scoparia* inbred lines averaged over two runs^a^ ^–^ ^e^.

Reproductive traits	Density (plants m^-2^)											
	1				85				170			
	SUS	CHES01	JOP01	Pooled	SUS	CHES01	JOP01	Pooled	SUS	CHES01	JOP01	Pooled
**Harvest index**	0.036 (0.003)	0.039 (0.003)	0.039 (0.003)	0.038a	0.026 (0.002)	0.025 (0.001)	0.027 (0.002)	0.026b	0.016 (0.001)	0.018 (0.001)	0.017 (0.002)	0.017c
**1000-seed mass (g)**	0.704 (0.05)	0.664 (0.05)	0.690 (0.04)	0.686a	0.516 (0.02)	0.487 (0.03)	0.498 (0.03)	0.500b	0.467 (0.02)	0.474 (0.02)	0.453 (0.02)	0.465b
**Seed viability (%)**	99 (1.36)	97 (1.34)	98 (1.80)	98a	99 (1.34)	98 (1.30)	100 (1.33)	99b	98 (1.32)	100 (1.39)	99 (1.35)	99c
**Radicle length (cm)**	1.90 (0.09)	1.87 (0.08)	1.93 (0.07)	1.90a	1.37 (0.08)	1.26 (0.07)	1.33 (0.09)	1.32b	1.33 (0.08)	1.20 (0.06)	1.30 (0.08)	1.27c

^a^ Values in parenthesis represent standard error of the mean.

^b^ Pooled means for each trait within a row with similar letters are not different based on Fisher’s protected LSD test at P < 0.05.

^c^ Harvest index was calculated as the ratio of total seed mass to aboveground biomass per plant.

^d^ Seed viability was determined as the percentage of total seeds that germinated plus those tested positive in tetrazolium chloride test.

^e^ Radicle length was recorded at 24 h after incubation of glyphosate-resistant (CHES01, JOP01) and glyphosate-susceptible (SUS) *K*. *scoparia* seeds in the germination assay.

#### Seed viability and radicle length

The interaction of experimental run by treatment was not significant (P = 0.564). Seed viability was not influenced by *K*. *scoparia* density, and ranged from 98 to 99% across the GR and SUS inbred lines (Table 3). Seeds produced by all kochia inbred lines across the three densities germinated ≥ 96% (data not shown). Progeny seedlings produced by GR or SUS plants grown under intraspecific densities of 85 and 170 plants m^-2^ had 30 and 33% less radicle lengths, respectively, compared with the seedlings obtained from the plants grown without competition (Table 3).

### Relationship of *EPSPS* genomic copy number and glyphosate resistance

**Fig 3:**
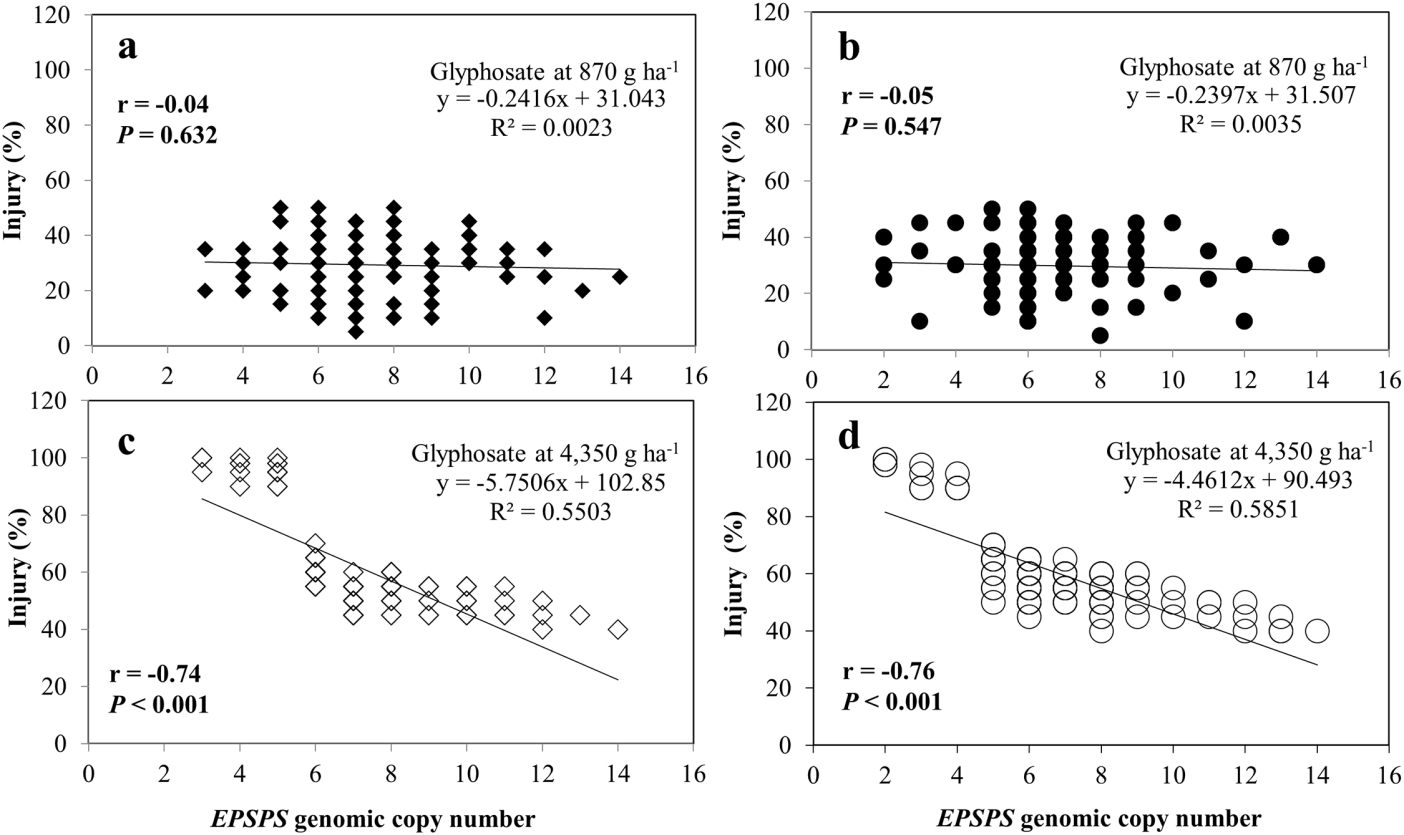
Relationship between *EPSPS* genomic copy number and percent injury of glyphosate-resistant (GR) *K*. *scoparia* inbred lines JOP01 treated with glyphosate at a) 870 and c) 4,350 g ha^-1^, and CHES01 treated with glyphosate at b) 870 and d) 4,350 g ha^-1^. Sample size was pooled from two experimental runs (n = 150). P value and Pearson’s correlation coefficient (*r*) between *EPSPS* genomic copy number and percent injury for each inbred line were estimated using PROC CORR in SAS.

In both experimental runs, none of the clones of SUS *K*. *scoparia* inbred line (with a single copy of the *EPSPS* gene) survived glyphosate applied at 870 or 4,350 g ha^-1^ (data not shown). In contrast, all clones of CHES01 and JOP01 inbred lines (with 2- to 14-fold amplification of the *EPSPS* gene) survived (< 55% injury) the 870 g ha^-1^ rate of glyphosate at 21 DAT (Fig 3a and 3b). However, at the 4,350 g ha^-1^ rate of glyphosate, survival of GR CHES01 or JOP01 inbred lines varied with the *EPSPS* gene copy number; some individuals survived with 40 to 70% injury and some were dead or had ≥ 90% injury at 21 DAT (Fig 3c and 3d). The Pearson’s correlation analyses revealed a strong relationship between the *EPSPS* genomic copy number and percent injury of GR CHES01 and JOP01 clones treated with glyphosate at 4,350 g ha^-1^ (Fig 3c and 3d). Furthermore, a negative linear relationship was observed between the *EPSPS* gene copy number and percent injury (Fig 3c and 3d). Only those GR CHES01 plants that had 5 or more copies of the *EPSPS* gene survived the 4,350 g ha^-1^ rate of glyphosate. For the GR JOP01 inbred line, at least 6 copies of the *EPSPS* gene were needed to survive this high rate of glyphosate.

## Discussion and Conclusions

*EPSPS* gene amplification confers glyphosate resistance in *K*. *scoparia* populations from northern and central Great Plains of the United States [14, 16, 27]. The higher copies of the *EPSPS* gene in GR *K*. *scoparia* correlated positively with the higher *EPSPS* transcription and EPSPS protein [14, 27]. Consequently, the EPSPS enzyme remains uninhibited [14, 16, 27], and enables the GR plants to survive lethal doses of glyphosate, up to 4,350 g ha^-1^ as evident from this research. Furthermore, the amplified copies of the *EPSPS* gene in GR *K*. *scoparia* did not affect other enzymes involved in the shikimate pathway [14]. In this research, we hypothesized that the tested GR *K*. *scoparia* populations might differ in growth and reproduction relative to the SUS population in the absence of glyphosate. In the absence of herbicide selection, fitness costs play an important role in preventing the fixation of novel herbicide resistance alleles and maintenance of genetic polymorphisms within weed populations [17, 18].

*K*. *scoparia* is a monoecious species, with numerous small inconspicuous flowers borne in the leaf axil of the whole plant. Additionally, the protogynous nature of flowering (stigma receptive before anthers dehisce) [3, 7] makes crossing efforts for creation of segregating F_2_ families from HS × HR crosses or development of near-isogenic lines, extremely time consuming and labor intensive. Therefore, an alternative approach of recurrent group selection [29–31] was used to generate the phenotypically uniform individuals from field-collected populations of GR and SUS *K*. *scoparia*, located in close proximity (2-km radius), and with similar cropping and herbicide use history. It is to be noted that two GR *K*. *scoparia* inbred lines were used in the study, and clones (shoot cuttings) were developed to confirm the *EPSPS* gene copies (mechanism of resistance) and glyphosate resistance of each plant (GR or SUS) utilized for the phenotypic characterization (growth and reproductive traits).

Results from this research indicate no relationship of *EPSPS* gene amplification (2 to 14 fold) with the growth or reproductive traits of the GR compared to the SUS *K*. *scoparia* inbred lines grown in an intraspecific competition gradient, in the absence of glyphosate. No differences in plant height and leaf area between the SUS (1 copy of *EPSPS* gene) and GR inbred lines (2 to 14 copies of *EPSPS* gene) when grown with or without intraspecific competition indicate that the inbred lines would be equally competitive in their ability to harvest light [21, 37]. The SUS and GR inbred lines accumulated similar amounts of aboveground biomass with or without competition. Consistent with our results, *EPSPS* gene amplification did not correlate with the vegetative growth traits in a GR compared to a glyphosate-susceptible (GS) *A*. *palmeri* biotype, in a fitness study conducted under greenhouse conditions [22, 23]. Harvest index quantifies the resource allocation of a plant to reproduction, and is an important determinant to study fitness costs associated with evolved herbicide resistance alleles [17, 18]. The results from our research indicated that the fecundity and relative allocation of resources to reproduction (harvest index) were similar across the GR and SUS inbred lines, irrespective of intraspecific densities. Also, in *A*. *palmeri*, up to 160-fold amplified copies of the *EPSPS* gene did not influence the inflorescence biomass of GR compared to GS plants [23]. Furthermore, no observed differences in seed mass between the GR and SUS *K*. *scoparia* indicate that the seedling vigor of the two GR inbred lines would be similar to that of the SUS inbred line [38].

This study also highlights that there is a positive correlation of *EPSPS* gene amplification with the glyphosate resistance levels in GR *K*. *scoparia* inbred lines, implying a greater resistance at the whole-plant level is associated with a greater *EPSPS* gene copy number. As few as 2 copies of the *EPSPS* gene conferred resistance to glyphosate at the field use rate (870 g ha^-1^) in the GR *K*. *scoparia* inbred lines. GR individuals with 2 to 4 copies of the *EPSPS* gene demonstrated ‘low level of resistance’ (survived the 870 g ha^-1^ rate of glyphosate, but failed to survive the 4,350 g ha^-1^ rate at 21 DAT). However, GR plants with 5 or more copies of the *EPSPS* gene showed ‘high level of resistance’ to glyphosate (survived the 4,350 g ha^-1^ rate of glyphosate at 21 DAT). Similarly, an increase in the glyphosate resistance level with an increase in the *EPSPS* gene copy number has also been reported in GR *K*. *scoparia* populations from Kansas [16, 39]. These results clearly indicate that regardless of evolution of glyphosate resistance in different locations, the mechanism and correlation of *EPSPS* copies with the level of resistance appears to be the same.

An additive effect of higher *EPSPS* gene copies on increased levels of glyphosate resistance has also been documented in *A*. *palmeri and Lolium perenne* ssp. multiflorum [13, 23]. Nevertheless, GR *A*. *palmeri* exhibited a greater magnitude of amplification of the *EPSPS* gene (2 to 160 fold); 21 copies of *EPSPS* were needed to survive 200 g ha^-1^ of glyphosate (low level of resistance), and 63 copies were needed to survive 2000 g ha^-1^ of glyphosate (highly resistant) [23]. In contrast, the results on GR *K*. *scoparia* suggest that a small increase in *EPSPS* gene copy number coupled with a small change in *EPSPS* expression (mediated by altered *EPSPS* promoter or enhanced activity of the transcription factor) may be sufficient to confer glyphosate resistance in plant species [14]. The results described here indicate that no resource tradeoff is necessary; overexpression of *EPSPS* in GR *K*. *scoparia* may well be within the metabolic capacity of the plant, which arguably is not surprising given the relatively small amounts of *EPSPS* protein involved [27].

It is expected that *EPSPS* gene copies in *K*. *scoparia* populations may increase with continued selection pressure from glyphosate [14, 16]. This together with no association of *EPSPS* gene copy numbers with the growth or reproduction may confer evolutionary advantages of the *EPSPS* gene amplification over other glyphosate resistance mechanisms [23]. The variation in the *EPSPS* gene copies among individuals in a GR inbred line of *K*. *scoparia* implies that the resistance polymorphism may contribute to the segregation of resistant individuals within a field population, differing in their ability to survive various glyphosate selective rates.

This research will aid in predicting the evolutionary trajectory of glyphosate resistance in *K*. *scoparia* in this region [17, 18]. A rapid development of glyphosate resistance in *K*. *scoparia* in GR or wheat-fallow cropping systems of this region is expected, especially when the glyphosate-selected trait confers a significant level of resistance [23]. Furthermore, wind-mediated tumble mechanism of seed dispersal coupled with pollen-mediated gene flow would ensure rapid movement of resistance alleles in *K*. *scoparia* populations [26, 27]. Importantly, this research highlights that GR *K*. *scoparia* individuals will likely persist in field populations even if the growers discontinue glyphosate use. No observed differences in growth and fecundity traits of GR vs. SUS *K*. *scoparia* may render the long-term management of GR *K*. *scoparia* even more challenging because periods of alternative weed control strategies may likely have less impact on reducing the frequency of GR alleles, compared to a scenario where the resistance allele endows a fitness cost [21, 40]. An integrated weed management program for *K*. *scoparia* should be implemented in GR crops or chemical fallow-based crop rotations that does not involve glyphosate [21]. Growers should make all possible efforts to prevent GR *K*. *scoparia* infestation in their production fields by utilizing tillage, effective soil-residual preemergence and alternative postemergence herbicide sites of action, and utilize mowing, cutting, or late-season herbicides to prevent seed set and any replenishment of the weed seed bank in the soil [26, 41, 42]. To further confirm the results reported in this study, resistance allele frequency or changes in the *EPSPS* gene copy numbers of GR *K*. *scoparia* should be assessed over multiple generations in the absence of glyphosate. Results obtained from our research should be further validated under field conditions to best simulate the environmental conditions in which fitness costs of HR alleles can be expressed [18]. Future research should evaluate the life history traits of GR *K*. *scoparia* under different levels of biotic and abiotic stresses, such as predation, disease, soil nutrient, temperature and moisture gradients, crop competition, and in the presence of glyphosate selection pressure.
