# Correction: Growth and Reproduction of Glyphosate-Resistant and Susceptible Populations of *Kochia scoparia*

**DOI:** 10.1371/journal.pone.0147779

**Published:** 2016-01-26

**Authors:** Vipan Kumar, Prashant Jha

There are formatting errors in Table 3 of the published article. Please see the correct Table 3 here.

**Table 3:** Effect of intraspecific competition on reproductive traits of glyphosate-susceptible (SUS) and glyphosate-resistant (CHES01 and JOP01) *K*. *scoparia* inbred lines averaged over two runs.^a^^-^^e^

Reproductive traits	Density (plants m^-2^)											
	1				85				170			
	SUS	CHES01	JOP01	Pooled	SUS	CHES01	JOP01	Pooled	SUS	CHES01	JOP01	Pooled
**Harvest index**	**0.036 (0.003)**	**0.039 (0.003)**	**0.039 (0.003)**	**0.038^a^**	**0.026 (0.002)**	**0.025 (0.001)**	**0.027 (0.002)**	**0.026^b^**	**0.016 (0.001)**	**0.018 (0.001)**	**0.017 (0.002)**	**0.017^c^**
**1000-seed mass (g)**	**0.704 (0.05)**	**0.664 (0.05)**	**0.690 (0.04)**	**0.686^a^**	**0.516 (0.02)**	**0.487 (0.03)**	**0.498 (0.03)**	**0.500^b^**	**0.467 (0.02)**	**0.474 (0.02)**	**0.453 (0.02)**	**0.465^b^**
**Seed viability (%)**	**99 (1.36)**	**97 (1.34)**	**98 (1.80)**	**98^a^**	**99 (1.34)**	**98 (1.30)**	**100 (1.33)**	**99^a^**	**98 (1.32)**	**100 (1.39)**	**99 (1.35)**	**99^a^**
**Radicle length (cm)**	**1.90 (0.09)**	**1.87 (0.08)**	**1.93 (0.07)**	**1.90^a^**	**1.37 (0.08)**	**1.26 (0.07)**	**1.33 (0.09)**	**1.32^b^**	**1.33 (0.08)**	**1.20 (0.06)**	**1.30 (0.08)**	**1.27^b^**

^a^ Values in parenthesis represent standard error of the mean.

^b^ Pooled means for each trait within a row with similar letters are not different based on Fisher’s protected LSD test at P < 0.05.

^c^ Harvest index was calculated as the ratio of total seed mass to aboveground biomass per plant.

^d^ Seed viability was determined as the percentage of total seeds that germinated plus those tested positive in tetrazolium chloride test.

^e^ Radicle length was recorded at 24 h after incubation of glyphosate-resistant (CHES01, JOP01) and glyphosate-susceptible (SUS) *K*. *scoparia* seeds in the germination assay.
